# Bone turnover and bone mineral density in HIV-1 infected Chinese taking highly active antiretroviral therapy –a prospective observational study

**DOI:** 10.1186/1471-2474-14-224

**Published:** 2013-07-30

**Authors:** Lixia Zhang, Yuanbo Su, Evelyn Hsieh, Weibo Xia, Jing Xie, Yang Han, Ying Cao, Yanling Li, Xiaojing Song, Ting Zhu, Taisheng Li, Wei Yu

**Affiliations:** 1 Department of Infectious Disease, Peking Union Medical College Hospital, Chinese Academy of Medicine Science, Beijing, China; 2 Department of Endocrinology, Peking Union Medical College Hospital, Chinese Academy of Medicine Science, Beijing, China; 3 Department of Radiology, Peking Union Medical College Hospital, Chinese Academy of Medicine Science, Beijing, China

**Keywords:** Acquired immunodeficiency syndrome (AIDS), Bone mineral density (BMD), Bone turnover marker (BTM), Highly active antiretroviral therapy (HAART)

## Abstract

**Background:**

Low bone mass and high bone turnover have been reported in HIV-infected individuals, both as a consequence of HIV infection itself, as well as from treatment with highly active antiretroviral therapy (HAART). The purpose of this study is to evaluate the impact of HAART on bone mineral density and bone turnover in HIV-1 infected Chinese patients.

**Methods:**

Forty HIV-1 infected patients were enrolled in this study; all patients were followed through 48 weeks, and 17 patients completed 96 weeks. Bone mineral density (BMD), procollagen type 1 N-terminal propeptide (P1NP), collagen type 1 cross-linked C-telopeptide (β-CTX), parathyroid hormone (PTH), and 25-OH vitamin D levels were measured at baseline, 48 and 96 weeks. Baseline measurements were compared with an age-, gender-, and BMI-matched healthy control population.

**Results:**

At baseline, raw BMD in the lumbar spine of HIV-1 infected patients was significantly lower than that of healthy controls (1.138 ± 0.112 g/cm^2^ vs. 1.195 ± 0.139 g/cm^2^, *p* = 0.047). During the first 48 weeks after initiating HAART, BMD of lumbar spine, femoral neck, and total hip decreased significantly in HIV-1 infected patients, with annual percent decline ranging from 1.78-3.28%. However, from week 48 to 96, BMD remained stable. Baseline levels of β-CTX (0.31 ± 0.16 ng/mL vs. 0.42 ± 0.19 ng/mL, *p* = 0.008) and P1NP (32.96 ± 14.00 ng/mL vs. 55.82 ± 26.87 ng/mL, *p* = 0.05) were lower in HIV-infected patients compared with controls, respectively. Both β-CTX and P1NP levels increased after onset of HAART until week 48, and remained elevated during the next 48 weeks. 25-OH vitamin D in HIV-infected patients was lower at baseline compared to healthy controls, but this difference was not statistically significant. PTH, however, was higher in HIV patients at baseline, and showed a significant increase throughout the study.

**Conclusions:**

Chinese adults with HIV-1 infection have low bone turnover prior to HAART as well as lower raw BMD of the lumbar spine compared with healthy controls, with further bone loss occurring following the initiation of HAART. The long-term clinical implications of these findings remain unclear at this time.

## Background

Low bone mass and high bone turnover have been reported in HIV-infected individuals, both as a consequence of HIV infection itself [1-4], as well as from treatment with highly active antiretroviral therapy (HAART) [5-7]. The rapid decline in bone mineral density (BMD) and rise in bone turnover markers (BTM) that has been observed in patients treated with HAART is especially pronounced during the first six months after initiation of HAART, and is followed by eventual stabilization of these parameters after one to two years [8-10]. Moreover, Vitamin D deficiency and secondary hyperparathyroidism may also be seen concurrently [11-15].

Limited reports have described the impact of HIV and HAART on BMD in HIV-infected Chinese. We previously observed lower BMD among a cross-sectional cohort of 62 HIV-infected patients compared to 20 non-infected controls [4]. No studies have been published regarding bone turnover among this population. It is possible that the bone metabolism of Chinese HIV-infected patients treated with HAART differs from that of other races. Yao et al. found that Chinese patients receiving HAART manifest a distinct pattern of lipodystrophy compared with Western populations [16]. As well, pharmacokinetic differences among Chinese HIV-1infected patients receiving HAART have been documented. Data from Wang et al. indicate that the ideal Nevirapine trough concentrations to achieve efficacy appears to be higher among Chinese HIV-1 infected individuals than the cutoff of 3.0 ug/ml suggested by previous cohort studies conducted in Caucasian patients [17,18].

The present study measures baseline BMD and bone turnover in a cohort of 40 HIV-infected individuals compared with 40 sero-negative healthy controls, and also examines the change in BMD, BTMs, parathyroid hormone (PTH) and 25-OH vitamin D in the HIV-1 infected patients during the 96 weeks after initiation of HAART.

## Methods

### Subjects

From April 2007 to March 2011, all adult patients (≥18 years of age) infected with HIV-1 and naive to ART presenting for care in the infectious disease clinic of Peking Union Medical College Hospital (PUMCH) were invited to participate in this study. Patients were excluded from the study if they had any of the following conditions: (1) significant renal, hepatic, thyroid or parathyroid dysfunction; (2) malignancies; (3) recent opportunistic infection; (4) a history of wasting syndrome; (5) intravenous injection drug use; (6) use of systemic glucocorticoids; (7) use of anti-osteoporotic agents; and (8) pregnant or nursing women.

A total of 201 patients were screened and of those, forty patients were both willing and met eligibility criteria for the study (35 males and 5 females). Healthy controls matched for age, gender, and BMI were recruited voluntarily from the general medicine clinic at PUMCH. All controls were tested and confirmed to be negative for HIV-1 at the time of enrollment. This study was approved by ethics committee of PUMCH, and written informed consent was obtained from each subject at the time of enrollment into the study.

After collection of baseline measurements, patients with HIV-1 infection were initiated on antiretroviral therapy with two nucleoside reverse transcriptase inhibitors (NRTIs) plus one non-nucleoside reverse transcriptase inhibitor (NNRTI), according to standard antiretroviral (ARV) regimens used in China. These regimens included Stavudine (D4T, 40 mg, twice daily) or Zidovudine (AZT, 300 mg, twice daily), paired with Lamivudine (3TC, 300 mg, once daily) and Nevirapine (NVP, 200 mg, twice daily for 14 days, followed by 200 mg once daily). All forty HIV-1 infected patients completed 48 weeks of follow-up, and 17 patients completed 96 weeks of follow-up.

### Procedures and measurements

Baseline and follow-up measurements: Upon entry into the study, weight and height was recorded for all participants. For all the patients at baseline and controls, bone mineral density was determined by dual X-ray absorptiometry (DEXA), serologic samples were taken and stored at -80°C until analysis for bone turnover markers, 25-OH vitamin D and PTH. In HIV-1 infected subjects, plasma viral load and peripheral lymphocyte subpopulation analysis were also evaluated. HIV-1 infected subjects were then followed prospectively and re-evaluated at weeks 48 and 96. At each of these time points, bone mineral density and serologic analysis was repeated.

Bone mineral density measurements: Bone mineral density of the total hip, femoral neck, and lumbar spine (the first to fourth lumbar vertebrae) was measured using the Lunar Prodigy Advance PA + 300388 (GE Healthcare, USA) (inter-assay CV for lumbar spine, femoral neck and total hip is 0.76%, 1.15% and 0.62% respectively). All measurements were performed in dorsal decubitus position using the same DEXA scanner, and results were interpreted using GE lunar software (enCORE version 10.50.086) by a single investigator.

Laboratory measurements: Electro-chemiluminescence immunoassay (Roche, modular analytics e 170, cobas e 601) was used to evaluate bone formation marker total procollagen type 1 N-terminal propeptide (P1NP; inter-assay and intra-assay coefficients of variation [CV] 3.8% and 2.9%, respectively) and the bone resorption marker serum collagen type 1 cross-linked C-telopeptide (β-CTX; inter-assay CV 7.6%, intra-assay CV 5.5%), PTH (inter-assay CV 3.4%, intra-assay CV 2.0%) and 25-OH vitamin D (inter-assay CV 13.1%, intra-assay CV 6.8%) .

HIV-1 testing of the control subjects was performed via ELISA (Wantai, HIV Ag-Ab 0496). For HIV-infected patients enrolled in the study, routine laboratory tests for peripheral lymphocyte subpopulation analysis and HIV RNA were performed at baseline and during follow-up. To determine the counts and proportion of CD4^+^T cells and CD8^+^T cells, peripheral lymphocyte subpopulation analysis was performed using whole blood samples and BD FACS canto (BD Bioscience, USA). HIV-1 plasma viral load was detected by Roche COBAS TaqMan (Roche, CA, USA2E2).

### Statistical analysis

Median and interquartile range (IQR) were calculated for CD4^+^T and CD8 ^+^T cell counts as well as viral loads and mean ± standard deviation calculated for other baseline variables. Baseline comparisons of categorical variables between the two study groups were performed using the Student’s *t*-test for independent samples. Repeated measures analysis was used to assess for changes in BMD, β-CTX, P1NP, PTH and 25-OH vitamin D over time among patients with HIV-1 infection. All statistical analysis was performed using the software package SPSS 16.0 (SPSS Inc., Chicago, IL, USA). Statistical significance was set at the level of 0.05 and all p-values were 2-tailed.

## Results

### Baseline characteristics

Forty HIV-1 infected, ART-naive patients were enrolled in the study, including 35 men (mean age 37.30 ± 10.11 years, range 20–55 years, mean BMI 22 ± 3.31 kg/m^2^) and five women (mean age 39.2 ± 9.26 years, range 31–54 years, BMI 24.56 ± 6.65 kg/m^2^). The study participants reflected the overall clinic population in terms of ethnicity (almost exclusively Han Chinese) and age distribution. However a greater proportion of the study population was male compared to the overall clinic population (87.5% v. 75.1%, *p* = 0.042). Forty age-, gender- and BMI-matched control subjects were enrolled and their baseline characteristics compared with the HIV-infected patients is shown in Table 1. The major route of transmission among HIV-1 infected patients was sexual intercourse (70%), with the remaining HIV-1 infections attributable to paid blood donation. At baseline, the median CD4^+^ T cell count in the HIV group was 137 (IQR 63–204) cells/μL, and the median plasma viral load was 4.42(IQR 3.59-4.86) log copies/mL. Therefore, the majority of the HIV-infected patients met diagnostic criteria for AIDS.

**Table 1:** Baseline characteristics

**Variables**	**Controls**	**HIV (+) Patients at baseline**	***p-value***
	**(n =** **40)**	**(n =** **40)**	
**Female/Male**	5/35	5/35	
**Age (mean years)**	37.19 ± 10.34	37.30 ± 9.92	0.756
**BMI (mean kg/m**^**2**^**)**	23.43 ± 3.35	22.32 ± 3.85	0.225
**Duration of HIV infection (mean years)**	NA	5.85 ± 3.07	
Viral load* (log10 copies/mL)	NA	4.42 (3.59-4.86)	
**CD4**^**+**^T cell count* (cells/μl)	NA	137 (63–204)	
**CD4**^**+**^**T cell percentage (%)**	NA	10.78 ± 6.84	
**CD8**^**+**^T cell counts* (cells/μl)	NA	773 (539–1027)	
**CD8**^***+***^**T cell percentage (%)**	NA	57.73 ± 9.77	
**BMD (mean g/cm**^**2**^**)**			
**Lumbar spine**	1.195 ± 0.139	1.138 ± 0.112	0.047
**Femoral neck**	1.035 ± 0.166	0.972 ± 0.136	0.069
**Total hip**	1.060 ± 0.126	1.015 ± 0.147	0.146
**BMD (mean Z score)**			
**Lumbar spine**	0.654 ± 1.077	0.511 ± 0.838	0.498
**Femoral neck**	0.382 + 1.189	0.255 ± 1.018	0.591
**Total hip**	0.377 ± 1.157	0.244 ± 1.102	0.604
**β-CTX (mean ng/mL)**	0.42 ± 0.19	0.31 ± 0.16	0.008
**P1NP (mean ng/mL)**	55.82 ± 26.87	32.96 ± 14.00	0.000
**PTH (mean pg/mL)**	22.92 ± 10.17	41.78 ± 16.11	0.000
**25-OH vitamin D (mean ng/mL)**	19.12 ± 9.00	15.93 ± 6.49	0.073

Illustrations for Table 1. *Values are expressed as median (interquartile range); BMI = weight (kg)/(height [m])^2^; *BMD* bone mineral density; lumbar spine: the first to fourth lumbar vertebrae; *P1NP* procollagen type 1 N-terminal propeptide; *β-CTX* Type I collagen cross-linked C-telopeptide; *PTH* parathyroid hormone.

In this study, all HIV-1 infected patients demonstrated a robust response to ART initiation, with undetectable HIV-1 RNA levels by week 48, and mean CD4^+^T cell counts of 242 (IQR 151–327) cells/μL at week 48 and 256(206–463) cells/μL at week 96.

### Change in BMD before and after HAART

There were no cases of osteoporosis or osteopenia in our study population at baseline or during the follow up period. However at baseline, the mean raw BMD in the lumbar spine of HIV-1 infected patients was significantly lower than that of healthy controls (1.138 ± 0.112 vs. 1.195 ± 0.139 g/cm^2^, *p* = 0.047), although this difference was not reflected in the BMD Z-score. There was no significant difference in mean raw BMD at the femoral neck and total hip compared with the controls, nor in the Z-scores (Table 1).

Raw BMD of the lumbar spine, femoral neck and total hip decreased significantly in HIV-1 infected patients from baseline to week 48 after initiating HAART (lumbar spine 1.138 ± 0.112 vs. 1.112 ± 0.113, *p* = 0.022; femoral neck 0.972 ± 0.136 vs. 0.940 ± 0.135, *p* = 0.000; total hip 1.015 ± 0.147 vs. 0.996 ± 0.142, *p* = 0.002). A significant decrease was also seen in the BMD Z-score at the femoral neck (0.255 ± 1.018 vs. -0.0125 ± 0.942, *p* = 0.014), but not at the lumbar spine or total hip. From week 48 to 96, BMD did not change significantly (Table 2).

**Table 2:** **Bone mineral density and laboratory results at baseline**, **48 and 96 weeks**

	**HIV-infected patients**			***p-value***^**#**^
**Variables**	**Baseline**	**Week 48**	**Week 96**	
**BMD (mean g/cm**^**2**^**)**				
**Lumbar spine**	1.138 ± 0.112	1.112 ± 0.113	1.120 ± 0.111	0.022
**Femoral neck**	0.972 ± 0.136	0.940 ± 0.135	0.942 ± 0.133	0.000
**Total hip**	1.015 ± 0.147	0.996 ± 0.142	0.996 ± 0.140	0.002
**BMD (mean Z score)**				
**Lumbar spine**	0.511 ± 0.838	0.288 ± 0.902	0.550 ± 0.691	0.053
**Femoral neck**	0.255 ± 1.018	-0.0125 ± 0.942	0.177 ± 0.831	0.014
**Total hip**	0.244 ± 1.102	0.105 ± 1.033	0.412 ± 1.004	0.164
**β-CTX (mean ng/mL)**	0.31 ± 0.16	0.41 ± 0.13	0.36 ± 0.12	0.000
**P1NP (mean ng/mL)**	32.96 ± 14.00	50.91 ± 21.97	49.53 ± 17.32	0.000
**PTH (mean pg/mL)**	41.78 ± 16.11	51.81 ± 15.96	65.06 ±20.59	0.000
**25-OH vitamin D (mean ng/mL)**	15.93 ± 6.49	15.04 ± 7.14	13.77 ± 7.16	0.343

Illustrations for Table 2. BMD: bone mineral density; lumbar spine: the first to fourth lumbar vertebrae; P1NP: procollagen type 1 N-terminal propeptide; β-CTX: Type I collagen cross-linked C-telopeptide; PTH: parathyroid hormone. ^#^*P*-*value* indicates comparison between baseline and week48.

Calculation of the annual percent change in BMD showed a decline of 2.104 ± 5.572% at the lumbar spine, 3.28 ± 4.37% at the femoral neck, and 1.78 ± 3.47% at the total hip from baseline to week 48. Percent change in BMD at all sites remained essentially stable from week 48 to week 96.

### Change in BTMs in HIV-1 infected patients treated with HAART

At baseline, the mean level of the bone formation marker P1NP was lower in HIV-1 infected patients than in the controls (32.96 ± 14.00 vs. 55.82 ± 26.87 ng/mL, respectively, *p* = 0.000). As well, the mean level of the bone resorption marker β-CTX was lower in HIV-1 infected patients compared with the controls (0.31 ± 0.16 vs. 0.42 ± 0.19 ng/mL, respectively, *p* = 0.008).

**Figure 1:**
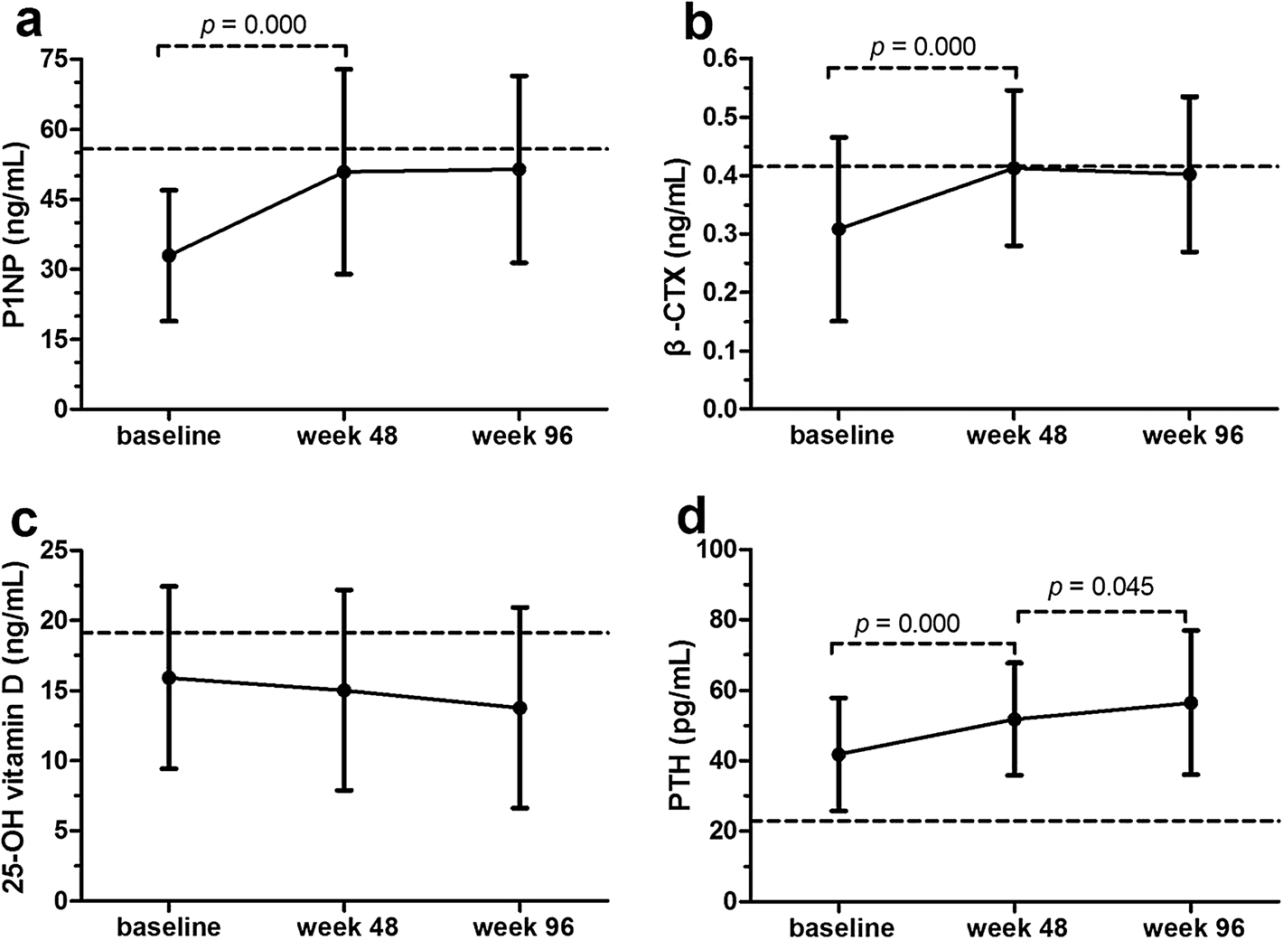
**The change of bone turnover markers, 25-OH vitamin D and PTH of HIV-infected patients at baseline, 48 weeks and 96 weeks. a **- The change of P1NP of HIV-infected patients and the controls; **b **- The change of β-CTX of HIV-infected patients and the controls; **c **- The change of 25-OH vitamin D of HIV-infected patients and the controls; **d **-The change of PTH of HIV-infected patients and the controls. (Dotted lines indicate the value in healthy controls, and solid lines indicate the value of HIV-1 infected patients. Abbreviations: P1NP, procollagen type 1 N-terminal propeptide; β-CTX, Type I collagen cross-linked C-telopeptide; PTH, parathyroid hormone).

From baseline to week 48, among HIV-1 infected patients taking HAART, both P1NP and β-CTX demonstrated a statistically significant increase compared with the baseline (Figure 1a and 1b), with an average percent increase of 54.44% for P1NP and 33.77% for β-CTX. This was followed by a plateau, with P1NP and β-CTX remaining stable from week 48 to week 96 (Figure 1a and 1b).

### 25-OH vitamin D and PTH levels in HIV-1 infected patients treated with HAART

25-OH vitamin D levels among HIV-1 infected patients in our study were low prior to HAART and remained low during follow-up. Mean 25-OH vitamin D levels in HIV-1 infected patients at baseline were lower than healthy controls, but this difference was not statistically significant (15.93 ± 6.49 ng/mL vs. 19.12 ± 9.00 ng/mL, respectively, *p* = 0.073). However, at week 48 and 96 after the initiation of HAART, mean 25-OH vitamin D levels in HIV-1 infected individuals were found to be significantly lower than that of baseline healthy controls (15.04 ± 7.14 ng/mL at week 48, *p* = 0.028 and 13.77 ± 7.16 ng/mL at week 96, *p* = 0.035) (Figure 1c).

Mean PTH levels were higher among HIV-1 infected patients at baseline than controls (41.78 ± 16.11 pg/mL vs. 22.92 ± 10.17 pg/mL, respectively, *p* = 0.000), and continued to rise during the follow-up period, reaching 51.81 ± 15.96 pg/mL at week 48 and 65.06 ± 20.59 pg/mL at week 96 (Figure 1d).

## Discussion

This is the first study that we know of reporting data on bone turnover in Chinese HIV-1 infected individuals. Our study demonstrated that prior to HAART, HIV-1 infected patients had lower BTM levels at baseline compared with non-infected controls. This finding differs from previously published studies, which have generally shown either no significant difference or higher BTM levels in untreated HIV-infected individuals compared with healthy controls [10,19,20].

One potential reason for this difference is that HIV-infected patients enrolled in our study had severe disease, with a median CD4^+^T cell count of 137 (IQR 63–204) cells/μL at baseline. Therefore at the time of diagnosis and HAART initiation, most patients in our study were already clinically diagnosed with AIDS. Studies by Ankrust et al. and Serrano et al. previously demonstrated lower bone formation in AIDS patients compared with non-AIDS HIV-infected patients and non-HIV infected patients [20,21]. The latter study demonstrated reduced bone formation and osteoclast numbers by histomorphometry in 22 HIV-infected patients and found histomorphometric parameters were more altered in patients with more severe disease. In terms of bone turnover, osteocalcin levels were lower in patients with more severe disease, however the authors were unable to detect a significant difference in bone resorption markers [21].

Furthermore, many studies in the general population have sought to characterize the role that race plays in bone metabolism. Epidemiologic and structural analyses have demonstrated differences in fracture rates, bone density, and bone architecture between populations of different races, including Chinese individuals compared to other races [22-25]. However understanding of the biology of these differences is still limited and studies investigating differences in biochemical markers of bone turnover have yielded mixed results [26,27]. In Chinese women tested at Peking Union Medical College Hospital, the average serum P1NP level is 43.78 ± 26.15 ng/mL (personal communication, Department of Endocrinology, PUMCH). By comparison, Eastell et al. reported average values of 38 ± 10.7 ng/mL in European white women tested by the same method [28]. Finally, as mentioned previously, the pharmacokinetics of therapeutic agents, including HAART, differs in Chinese patients compared with Western populations [17], therefore the magnitude of drug-specific effects of antiretrovirals on bone may be different. Further investigation into the specific impact of HAART on bone metabolism in Chinese HIV-infected patients is warranted.

With regards to the subsequent changes in bone turnover markers after initiation of HAART, in our study, levels of β-CTX and P1NP increased significantly during the first 48 weeks and stabilized from week 48 to 96, which is consistent with previously published studies [19,29,30]. These findings follow an inverse pattern compared with our BMD data, which showed a lower baseline raw BMD at the lumbar spine in patients with HIV-1 infection prior to HAART compared with healthy controls, followed by a decline in raw BMD in the lumbar spine, femoral neck, and total hip during the first 48 weeks of HAART, and subsequent stabilization between 48 to 96 weeks. The inverse correlation between BTM and BMD has been documented previously and studies have shown that in high turnover states such as menopause, this association becomes particularly strong [31,32]. Previous studies in HIV-infected patients have also demonstrated this inverse relationship [19,33].

Our BMD findings support the results of other studies from the literature demonstrating a decline in BMD among HIV-infected patients after initiation of HAART that is most pronounced within the first year [9,34,35]. Although our study primarily found statistically significant differences in the raw BMD, and only found a statistically significant decline in Z-score at the femoral neck, we believe that this may be related to the relatively young age of our study population and small sample size. Indeed, the annual percent change from baseline during the first year after initiation of HAART in our study is consistent with the magnitude of change that has been observed among HIV-infected patients initiating HAART in prior studies [34,36,37]. Finally, patients in our study were treated with zidovudine- and stavudine-based regimens. Although these are known to be associated with bone loss among HIV-patients, the most profound effects have been seen with Tenofovir and protease inhibitors [5,7,37-40]. In 2012 Tenofovir was made available in China as first-line therapy for HIV infected patients, therefore it will be increasingly important for future studies evaluating the impact of HAART on bone disease among Chinese HIV-infected patients to include those treated with Tenofovir-based regimens.

In our study, the mean level of 25-OH vitamin D in all groups was consistent with vitamin D deficiency (< 20 ng/mL). Levels of vitamin D in HIV-1 infected patients at baseline were lower compared with healthy controls, although this difference was not statistically significant, perhaps due to the limited sample size. There was a corresponding significant elevation in PTH levels in HIV-infected patients naive to HAART compared with healthy controls, which continued to increase with continued treatment. Prior studies in HIV-infected populations have also documented low vitamin D levels, and we suspect that larger scale studies will confirm lower vitamin D levels in Chinese HIV-infected patients compared with the healthy population [14,41].

This degree of vitamin D deficiency in Chinese HIV-infected patients was previously unrecognized and has extraskeletal implications as well. Haug et al. previously demonstrated an association between low vitamin D and low CD4^+^T cells counts in HIV infected patients [42], and a recent study showed that deficiency of vitamin D may impair recovery of CD4^+^T cells [43,44]. Thus vitamin D supplementation may not only be beneficial to bone metabolism, but may also help to improve immune reconstitution.

Our study has a few limitations. First, the sample size was small, in particular in the 96 week follow-up group, prohibiting subgroup analyses and making it difficult to draw conclusions regarding long-term effects. However, we were able to assess statistically significant differences in BMD and BTMs between HIV-infected patients at baseline and the control group. Furthermore, the plateaus in BMD and BTM observed from week 48 to week 96 are consistent with other published studies. Second, intravenous drug users were excluded from this study; however, if anything, we would expect this to lead to underestimation of the true bone mineral density among HIV-infected patients. Third, we did not include measurement of serum calcium, albumin and phosphorus, important indicators of bone metabolism that would have provided a more complete laboratory assessment of underlying bone status in our study population. Finally, data was not gathered regarding specific HAART regimen, tobacco history, or alcohol consumption factors, which may impact the underlying bone mineral density of individual subjects in our study. More detailed studies are needed to clarify the underlying bone metabolism in Chinese HIV-infected patients as well as the long-term impact of HAART in this population, given the biological and environmental differences between Chinese patients and Western populations.

## Conclusions

Chinese adults with HIV-1 infection have low bone turnover prior to HAART as well as lower BMD at the lumbar spine compared with healthy controls, with further bone loss occurring following the initiation of HAART. Vitamin D insufficiency and deficiency with associated secondary hyperparathyroidism are highly prevalent in this population. Further research is needed to elucidate the implications of these findings for screening, prevention and management of bone loss and vitamin D deficiency in this population.

## Competing interests

The authors declare that they have no competing interests.

## Authors’ contributions

The authors have contributed in the following ways: TL, WY and WX provided concept/research design. Lixiazhang, Yuanbo Su, Jing Xie, Yang Han, Ying Cao, Yanling Li, Xiaojing Song, Ting Zhu provided data collection, Lixia Zhang, Yuanbo Su, Evelyn. Hsieh and Weibo Xia provided data analysis and manuscript writing. All the authors agreed on the final content of the manuscript. All authors read and approved the final manuscript.

## Pre-publication history

The pre-publication history for this paper can be accessed here:

http://www.biomedcentral.com/1471-2474/14/224/prepub
